# PLGA-modified Syloid^®^-based microparticles for the ocular delivery of terconazole: in-vitro and in-vivo investigations

**DOI:** 10.1080/10717544.2022.2092239

**Published:** 2022-07-15

**Authors:** Nada Zaghloul, Azza A. Mahmoud, Nermeen A. Elkasabgy, Nada M. El Hoffy

**Affiliations:** a Department of Pharmaceutics and Pharmaceutical Technology, Faculty of Pharmacy, Future University in Egypt, Cairo, Egypt; b Department of Pharmaceutics and Industrial Pharmacy, Faculty of Pharmacy, Cairo University, Cairo, Egypt

**Keywords:** Terconazole, Syloid^®^, Mesoporous silica, PLGA, Ocular delivery

## Abstract

The eye is an invulnerable organ with intrinsic anatomical and physiological barriers, hindering the development of a pioneer ocular formulation. The aim of this work was to develop an efficient ocular delivery system that can augment the ocular bioavailability of the antifungal drug, terconazole. Mesoporous silica microparticles, Syloid^®^ 244 FP were utilized as the carrier system for terconazole. Preliminary studies were carried out using different drug:Syloid^®^ weight ratios. The optimum weight ratio was mixed with various concentrations (30 and 60%w/w) of poly (lactic-co-glycolic acid) (PLGA), ester or acid-capped and with different monomers-ratio (50:50 and 75:25) using the nano-spray dryer. Results revealed the superiority of drug:Syloid^®^ weight ratio of 1:2 in terms of yield percentage (Y%), SPAN and drug content percentage (DC%). Furthermore, incorporation of PLGA with lower glycolic acid monomer-ratio significantly increased Y%. In contrast, increasing the glycolic acid monomer-ratio resulted in higher DC% and release efficiency percentage (RE%). Additionally, doubling PLGA concentration significantly reduced Y%, DC%, drug loading percentage (DL%) and RE%. Applying desirability function in terms of increasing DC%, DL% besides RE% and decreasing SPAN, the selected formulation was chosen for DSC, XRD and SEM investigations. Results confirmed the successful loading of amorphized terconazole on PLGA-modified Syloid^®^ microparticles. Moreover, pharmacokinetic studies for the chosen formulation on male Albino rabbits’ eyes revealed a 2, 6.7 and 25.3-fold increase in mean residence time, C_max_ and AUC_0–24_-values, respectively, compared to the drug suspension. PLGA-modified Syloid^®^ microparticles represent a potential option to augment the bioavailability of ocular drugs.

## Graphical Abstract

**Figure:**
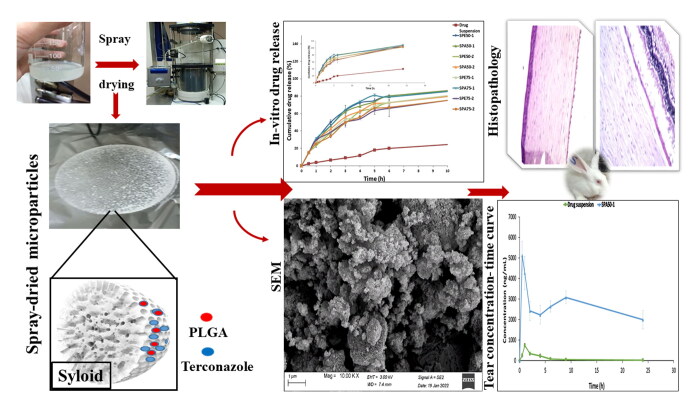

## 1. Introduction

The eye is the most delicate and complicated organ in the body. It is easily influenced by different chronic diseases such as cataract, glaucoma, dry eye, and several infectious diseases from bacterial, viral or fungal sources (Gupta et al., 2013). The most common and challenging among them is the fungal contamination which is mainly caused by the Candida species (Foster, 1992). Although they are responsible for most of the infections, yet, they are still challenging being resistant to the majority of the commonly used antifungal drugs (Patel et al., 2013).

Topical administration, mostly in the form of eye drops, is employed to treat anterior segment diseases. The location of action for most topically administered medications is usually the cornea, conjunctiva, sclera, and other anterior segment tissues such the iris and ciliary body (anterior uvea). The bioavailability of topical preparations is hampered by precorneal variables and anatomical barriers after application. Precorneal variables include solution drainage, blinking, tear film, tear turnover, and induced lacrimation (Jaeger & Tasman, 2009). Tear film, whose composition and amount are indicators of a healthy ocular surface, provide the initial barrier due to its high turnover rate (Mannermaa et al., 2006; Gaudana et al., 2010; Ding et al., 2018).

A triazole ketal derivative, terconazole proved its efficacy on a various range of yeasts and mycelium-forming fungi upon its *in-vivo* investigations. It acts as a powerful antifungal agent, preventing the yeast from morpho-genetically transforming into the highly infectious filamentous form of *Candida albicans* known as pseudomycelium. Additionally, it is highly active in topical treatment of numerous experimental models of dermatophytosis and candidosis (Heeres et al., 1983). Terconazole is white to almost white powder with a molecular weight of 532.47 Da. It has a log P and Pka of 5.37 and 8, respectively. Unfortunately, its poor aqueous solubility limits its ocular application. The literature sheds the light on several studies investigating the use of terconazole for ocular delivery applying various formulation techniques, including the fabrication of silica/chitosan nanoparticles (Zaghloul et al., 2022) and cationic polymeric nanoparticles (Mohsen, 2022).

Mesoporous silica has proved its potentiality to improve the poor aqueous solubility of many pharmaceutical moieties (Choudhari et al., 2014). As a drug carrier system, mesoporous silica can be loaded with drugs utilizing different techniques among which are the solvent immersion employing organic solvents as well as incipient wetness impregnation or melting in (Xu et al., 2013). Moreover, the safety and biocompatibility of the ocular use for mesoporous silica have been assessed (Sun et al., 2020). Amongst the pre-synthesized mesoporous silica carriers that are gaining much attention these days are the Syloids^®^. Syloid^®^ 244 FP is a micronized (diameter 2.5–3.7 μm) artificial amorphous silica particles with highly developed network of non-ordered mesopores, that provide the advantage of having large surface area (Limnell et al., 2011). The pharmaceutical roles of these silicas as excipients include their use as glidants, adsorbents, anti-tacking and desiccant agents, carriers for active pharmaceutical ingredients as well as thickening and gelling agents (Kinnari et al., 2011; Chaudhari & Gupte, 2017; Donnadio et al., 2019).

An extensive amount of research has been carried out on drug delivery using biodegradable polymers since being used as bioresorbable surgical devices nearly thirty years ago. Among all the biodegradable biomaterials, poly lactic-co-glycolic acid (PLGA) has demonstrated enormous potential as a drug delivery carrier and tissue engineering scaffold upon application. PLGA is a family of FDA-approved biodegradable polymers that are highly biocompatible with reliable physical characteristics (Jain, 2000; Ruhe et al., 2003; Bouissou et al., 2006; Makadia & Siegel, 2011; Wang et al., 2021). PLGA is made up of various ratios of lactic and glycolic acids, resulting in a wide range of physicochemical properties that are dependent on this lactic/glycolic acid ratio. The selection of the appropriate PLGA type and concentration during designing the formulation is critical for successful drug delivery (Mu & Feng, 2003). Moreover, PLGA has a proven safety profile in ocular drug delivery (Agnihotri & Vavia, 2009; Bertram et al., 2009) ensuring its safety onto the ocular tissues (Gupta et al., 2010, 2011).

Spray drying is gaining popularity as a gentle, one-step, continuous, and scalable method for drying liquids into dry powder to be used in different routes of administration (Mahmoud et al., 2018; Maged et al., 2020). It enables the drying of particles with controlled size and shape in the field of particle engineering (Schmid et al., 2011; Arpagaus, 2012). In addition, it allows for drug loading into a matrix, resulting in a stable final product (Ruffel et al., 2020), with amorphous characteristics due to the fast solvent evaporation (Vogt et al., 2008) as well as favorably spherical particles which have small size and narrow particle size distribution (Takeuchi et al., 1987, 2004).

In the following study, terconazole is loaded on the pre-synthesized mesoporous Syloid^®^ 244 FP and embedded with PLGA to improve and sustain the release profiles of the drug. The prepared formulations were characterized for their yield percentage, particle size, SPAN, drug content percentage, drug loading percentage and *in-vitro* drug release. The obtained data was statistically analyzed utilizing SPSS^®^ software. The optimum formulation was further investigated through differential scanning calorimetry and X-ray diffraction. Additionally, the morphological examination using scanning electron microscope was performed. Finally, the bioavailability of the optimum prepared formulation in tears was assessed in male Albino rabbits.

## 2. Materials and methods

### 2.1. Materials

Terconazole was provided by Minapharm Pharmaceuticals (Cairo, Egypt). Syloid^®^244 FP was received as a gift sample from W.R. Grace & Co. (St. Neots, UK). Poly(D, L-lactic/glycolic acid); PLGA with a lactide to glycolide ratio of 50:50, end-capped with acid terminal and an inherent viscosity of 0.2 dL/g (Purasorb^®^ PDLG 5002 A); PLGA with a lactide to glycolide ratio of 50:50, end-capped with ester terminal and an inherent viscosity of 0.2 dL/g (Purasorb^®^ PDLG 5002); PLGA with a lactide to glycolide ratio of 75:25, end-capped with acid terminal and an inherent viscosity of 0.22 dL/g (Purasorb^®^ PDLG 7502 A) and PLGA with a lactide to glycolide ratio of 75:25, end-capped with ester terminal and an inherent viscosity of 0.22 dL/g (Purasorb^®^ PDLG 7502) were kindly provided by Corbion (Amsterdam, Netherlands). Acetonitrile (HPLC grade), sodium chloride, sodium dihydrogen phosphate monohydrate and disodium hydrogen phosphate heptahydrate were purchased from El-Nasr Pharmaceutical Chemicals (Cairo, Egypt). Dialysis tubing cellulose membrane (molecular weight cutoff 12000-14000 g/mole) was procured from Sigma Aldrich (Saint louis, MO, USA). The rest of chemicals and solvents were of analytical grade and were utilized as received. Water was used as deionized, bi-distilled water.

### 2.2. Methods

#### 2.2.1. Preparation of nano-spray dried terconazole-loaded Syloid^®^ microparticles

Preliminary studies were performed to assure the proper terconazole: Syloid^®^ weight ratio to be used for further studies as shown in Table 1. Different weight ratios of 2:1, 1:1, 1:2, 1:4 and1:6 (w/w) terconazole: Syloid^®^ were prepared by completely dissolving accurate amounts of terconazole (5 mg/mL) in 10 mL acetonitrile under sonication (Water bath sonicator, Elma S30H, Singen, Germany) for 2 min. Subsequently, Syloid^®^ was dispersed into the previously prepared drug solution under sonication for another 3 min to ensure its good dispersion in the drug solution. Drug-loaded microparticles were formed by spraying the prepared dispersion through a nano-spray dryer (Nano Spray Dryer B-90; Buchi Labortechnik, Flawil, Switzerland) operated using nitrogen gas which was monitored for its oxygen content (less than 4%) by the inert loop B-295 system. The flow rate of nitrogen was adjusted at 100 L/min. The dispersion was sprayed using a nozzle size of 7.0 μm at inlet and outlet temperatures of 95 °C and 40 °C, respectively. Eventually, the spray-dried microparticles were collected and stored in closed containers pending further investigations.

**Table 1.** Composition and characterization of terconazole-loaded Syloid^®^ microparticles during preliminary studies.

Composition	Characterization						
**Terconazole : Syloid^®^** **weight ratio (w/w)**	**Yield** **(Y; %)**	Particle size (PS; µm)	SPAN	**Drug content** **(DC; %)**	Drug loading (DL; %)	**Q_0.5_** **(%)**	**Release efficiency** **(RE; %)**
**1:1**	33.00 ± 1.41	1.82 ± 0.19	1.42 ± 0.01	42.35 ± 0.93	20.53 ± 1.37	30.32 ± 0.15	64.88 ± 1.08
**1:2**	33.50 ± 0.71	1.89 ± 0.14	1.29 ± 0.23	73.86 ± 0.91	22.75 ± 0.64	28.42 ± 0.53	63.76 ± 1.99
**1:4**	30.69 ± 0.61	1.90 ± 0.13	1.29 ± 0.08	72.13 ± 0.74	15.20 ± 0.57	28.34 ± 0.09	63.51 ± 0.94
**1:6**	32.13 ± 2.24	1.94 ± 0.11	1.34 ± 0.18	73.98 ± 1.45	9.75 ± 1.06	28.28 ± 0.09	63.90 ± 0.15

* **Each formulation was prepared using 5 mg/mL terconazole dissolved in 10 mL acetonitrile**.

**Abbreviations; SPAN: width of the size distribution, Q_0.5_: percentage drug released after 0.5 h**.

#### 2.2.2. Characterization of the nano-spray dried terconazole-loaded Syloid^®^ microparticles

##### 2.2.2.1. Nano-spray drying process yield percentage determination (Y%)

The yield % of the collected spray-dried microparticles was computed using the following equation.

$$\mathrm{Yield}\;(\%)=\frac{\mathrm{Recovered}\;\mathrm{microparticles}\;\mathrm{weight}\;(\mathrm{mg})}{\mathrm{Total}\;\mathrm{initial}\;\mathrm{solids}\;\mathrm{weight}\;(\mathrm{mg})}\times 100$$

##### 2.2.2.2. Particle size (PS) and SPAN determination

Particle size as well as SPAN (width of the size distribution) values were characterized using the MasterSizer 2000 (Malvern Instruments Ltd, Worcestershire, UK). SPAN was calculated using the equation:

$$\mathrm{SPAN}\;=\frac{D(0.9)-D(0.1)}{\;D(0.5)}$$

where, D(0.9), D(0.1) and D(0.5) are the spherical equivalent volume diameters of microparticles that are below 90%, 10%, and 50% of the cumulative distribution, respectively.

##### 2.2.2.3. Percentage drug content (DC%) and drug loading (DL%) determination

All the prepared formulations were weighed and then add to acetonitrile toinvestigate the amount of loaded drug spectrophotometrically (UV-spectrophotometer, Shimadzu 1800 UV, Shimadzu, Kyoto, Japan) at the predefined ℷ_max_ (245.2 nm) after filtration through Millipore membrane filter (0.45 µm pore size). The following equations were used to compute the percentage drug content and drug loading:

$$\mathrm{Drug}\;\mathrm{content}\%=\frac{\mathrm{Actual}\;\mathrm{drug}\;\mathrm{amount}}{\;\mathrm{Theoritical}\;\mathrm{drug}\;\mathrm{amount}}\times 100$$

$$\mathrm{Drug}\;\mathrm{loading}\%=\frac{\mathrm{Drug}\;\mathrm{content}\;(\mathrm{mg})}{\;\mathrm{Weight}\;\mathrm{of}\;\mathrm{prepared}\;\mathrm{microparticles}\;(\mathrm{mg})}\times 100$$

##### 2.2.2.4. In-vitro drug release studies

The dialysis bag method was used to investigate the release pattern for the drug from the produced microparticles. Briefly, a specific weight of the prepared microparticles equivalent to 2 mg drug was carefully weighed and suspended in 1 mL isotonic phosphate buffer solution (pH 7.4) then instilled into a dialysis bag (representing the donor compartment). The filled dialysis bags were submerged in 20 mL isotonic phosphate buffer solution (pH 7.4) in glass bottles representing the receptor compartment to achieve sink conditions. In an incubation shaker (IKA KS 4000, Staufen, Germany), all the glass bottles were shaken at 34 °C ± 0.5 °C (Maged et al., 2016) at a rate of 160 strokes per minute. At predetermined times, 3 mL of the release medium were sampled and replaced by fresh medium to preserve the sink conditions. The withdrawn samples were analyzed for its drug content spectrophotometrically at 245.2 nm. Samples were compared by calculating the cumulative percentage of drug released at 0.5 h (Q_0.5_) and release efficiency percentage (RE%). The RE% was calculated from the area under the release curve (AUC) at time t using the following equation:

$$\mathrm{RE}\%=\frac{\int_{0}^{t}y\;\times \mathrm{dt}}{y_{100}\;\times 100}\;\times 100$$

Where, y is the amount of drug released at time t, y_100_ represents 100% drug release, and T is the total time of the release study.

#### 2.2.3. Preparation of modified microparticles

The desirability function was applied to the obtained results to choose the best drug: Syloid^®^ weight ratio for further investigations. The target criteria were maximizing yield percentage, drug content percentage, drug loading percentage and the release efficiency percentage and minimizing SPAN and Q_0.5_.

The drug: Syloid^®^ weight ratio with the highest overall desirability was further modified by adding different concentrations (30 and 60% w/w) of ester or acid terminal PLGA (Table 2) to obtain eight modified formulations. For preparing the modified formulations, the same steps mentioned under Section 2.2.1. were followed but with minor modification where the added PLGA was pre-dissolved in the drug/Syloid^®^ dispersion prior to the spray drying process.

**Table 2.** Compositions and characterization of terconazole PLGA-modified Syloid*^®^* microparticles.

**Formulation** **Code**	Composition					Characterization				
	PLGA monomers ratio	PLGA terminal type	**PLGA concentration** **(% w/v)**	**Yield** **(Y; %)**	Particle size (PS; µm)	SPAN	**Drug content** **(DC; %)**	Drug loading (DL; %)	**Q_0.5_** **(%)**	**Release efficiency** **(RE; %)**
**SPE_50_-1**	**50:50**	**Ester**	**30**	43.79 ± 2.22	1.780 ± 0.072	1.430 ± 0.014	56.26 ± 0.35	15.33 ± 0.52	15.13 ± 0.47	82.73 ± 0.36
**SPA_50_-1**	**50:50**	**Acid**	**30**	42.71 ± 0.77	1.763 ± 0.119	1.395 ± 0.014	59.31 ± 0.08	17.14 ± 0.97	15.69 ± 0.20	83.31 ± 0.86
**SPE_50_-2**	**50:50**	**Ester**	**60**	29.28 ± 1.02	1.711 ± 0.132	1.515 ± 0.014	54.88 ± 0.53	12.31 ± 0.94	15.11 ± 0.11	77.03 ± 0.80
**SPA_50_-2**	**50:50**	**Acid**	**60**	36.80 ± 1.15	1.734 ± 0.086	1.52 ± 0.0141	53.35 ± 0.57	12.39 ± 0.57	15.41 ± 0.52	79.14 ± 0.74
**SPE_75_-1**	**75:25**	**Ester**	**30**	48.18 ± 1.36	1.816 ± 0.13	1.443 ± 0.014	52.12 ± 0.19	14.07 ± 0.63	15.13 ± 0.49	79.32 ± 0.27
**SPA_75_-1**	**75:25**	**Acid**	**30**	54.91 ± 0.13	1.758 ± 0.074	1.423 ± 0.014	49.34 ± 0.47	13.28 ± 0.47	15.28 ± 0.07	84.57 ± 0.08
**SPE_75_-2**	**75:25**	**Ester**	**60**	43.56 ± 1.70	1.752 ± 0.022	1.569 ± 0.014	50.12 ± 0.18	11.51 ± 0.65	15.85 ± 0.17	73.12 ± 0.03
**SPA_75_-2**	**75:25**	**Acid**	**60**	31.57 ± 1.56	1.7145 ± 0.15	1.537 ± 0.014	47.23 ± 0.39	11.36 ± 0.08	15.13 ± 0.59	75.03 ± 0.74

* **Each formulation was prepared using 5 mg/mL terconazole dissolved in 10 mL acetonitrile**.

**Abbreviations; SPAN: width of the size distribution, Q_0.5_: percentage drug released after 0.5 h**.

#### 2.2.4. Characterization of the modified formulations

The modified formulations were characterized for their Y%, PS, SPAN, DC%, DL% and the obtained release data as previously mentioned. Again, the desirability function was applied to select the best modified formulation (optimized) for further *in-vitro* and *in-vivo* investigations. The same target criteria as mentioned previously were followed.

##### 2.2.4.1. Differential scanning calorimetry study (DSC)

The optimized formulation thermal behavior was investigated using differential scanning calorimeter (DSC-50, Shimadzu, Japan). DSC investigations were carried out on terconazole, PLGA with acidic terminal and monomers’ ratio of 50:50, Syloid^®^, the optimized formulation, physical mixture, as well as the PLGA-free analogues of both the formulation and its physical mixture. The process involved heating a precisely weighed sample (5 mg) encased in an aluminum pan at a scanning rate of 10^°^ C/min over a temperature range of 30 to 400^°^ C. Dry nitrogen gas served as the carrier pumped at a flow rate of 25 mL/min.

##### 2.2.4.2. X-ray diffraction study (XRD)

X-ray diffraction patterns of the selected optimized formulation along with its individual components, terconazole, PLGA with acidic terminal and monomers’ ratio of 50:50, Syloid^®^ and its physical mixture were assayed. In addition, the optimized formulation PLGA-free analogue and its physical mixture were investigated. The samples were exposed to Ni filtered Cu K_α_ radiation with voltage level of 45 kV and current of 40 mA using Diano X-ray diffractometer apparatus (USA). The used scanning rate was 2^°^/min over diffraction angle (2θ) range of 3-70^°^.

##### 2.2.4.3. Scanning electron microscopy (SEM)

The morphology and surface properties of the optimized formulation along with its components including the drug, Syloid^®^ and PLGA-free analogue were examined by a scanning electron microscope (Quanta FEG250, Hillsboro, Oregon, USA). The SEM was set to 20 kV. Gold sputtering with a Poloron DC (sputtering unit) operating at 1.4 kV and 18-20 mA was used to conduct the samples.

##### 2.2.4.4. Biological evaluation of the modified formulation

###### 2.2.4.4.1. Gamma sterilization 

For sterilization purposes, the modified formulation was exposed to gamma radiations using a gamma irradiator (Gammacell 1000; Best Theratronics, Ontario, Canada). To avoid temperature rises during the gamma-irradiation process, the vial containing the sample was originally placed in a polyurethane container filled with dry ice before the sterilization process. This precautionary measure was taken to avoid particle aggregation.

###### 2.2.4.4.2. Histopathological studies 

The safety of the selected formulation on the long-term ocular usage was evaluated by performing histopathological studies. Three male Albino rabbits (weighing 2.5-3.0 ± 0.25 kg) were given three repetitive doses of the optimized formulation (equivalent to 1 mg/mL terconazole suspended in isotonic phosphate buffer pH 7.4) in the right eye on daily basis for one week, while the left eye served as a control (untreated). On the seventh day, the rabbits were decapitated under mild anesthesia. The eyeballs were then detached, cleaned in saline solution, and immediately fixed in a 10% formal saline solution for 24 h. After fixation, dehydration was carried out using serial dilutions of ethyl alcohol. The dehydrated samples were cleared in xylene and embedded in paraffin at 56 °C in hot air oven for 24 h. Paraffin blocks were cross sectioned at a thickness of 4 microns. The acquired cross sections were mounted on glass slides, de-paraffinized, and stained with hematoxylin and eosin for routine observation under a light electric microscope (Leica Imaging Systems Ltd, Cambridge, England) (Baydoun et al., 2004).

###### 2.2.4.4.3. Pharmacokinetic study

####### 2.2.4.4.3.1. Study design 

A parallel design was adopted for the evaluation of the bioavailability of the optimized formulation in tears for male Albino rabbits compared to the free drug suspension.

The sterilized chosen formulation as well as the drug suspension equivalent to 1 mg/mL terconazole were suspended in isotonic phosphate buffer pH 7.4. The study design was approved by the Research Ethics Committee, Faculty of Pharmacy, Future university in Egypt, Cairo, Egypt (FPSPI 14/101). For the investigation, sixteen healthy male Albino rabbits weighing 2.5-3.0 ± 0.25 kg were randomly categorized into two groups. Each group was divided into two subgroups (*n* = 4). Each group received a single ocular dose of either the tested formulation or drug suspension. A volume of 50 µL of the investigated samples was instilled into the conjunctival sac of the eye using a micropipette. No animals were excluded from the study.

####### 2.2.4.4.3.2. Tear film sampling 

Tear film samples were taken to assess the amount of the drug present at the investigated time intervals. This was done by inserting three sterile filter paper disks (6 mm in diameter) under the rabbit’s lower eyelid and leaving it for 1 min before removal. This step was repeated on new rabbits (*n* = 4) at each of the investigated time intervals; 0.5, 1, 2, 4, 6, 9, and 24 h. During the sampling process, special care was taken to prevent irritating the eyelid margin. The collected disks were soaked in 100 µL ethanol in Eppendorf tubes followed by storage at −20°C until analysis.

####### 2.2.4.4.3.3. Chromatographic conditions 

The concentration of terconazole was determined in collected rabbits’ tear film using a validated liquid chromatography (LC-20AT, Shimadzu, Kyoto, Japan) equipped with triple quadrupole mass spectrometer (API 4000, AB Sciex Instruments, Framingham, MA, USA). Dapoxetine was utilized as an internal standard. The mobile phase consisted of 0.1%v/v formic acid in 80:20 volume ratio of acetonitrile to H_2_O and its pH value was adjusted at 8.5. Exactly 15 μL of the sample were injected into the column and the elution was operated at flow rate of 1.5 mL/min. The separation was performed on a C_18_ reversed phase analytical column (250 mm × 4.6 I.D. mm, particle size 5 μm, Berlin, Germany) and the drug was detected spectrophotometrically at 220 nm.

The pharmacokinetic parameters of terconazole were calculated using WinNonlin^®^ software (Version 3, Scientific Consulting Inc., Cary, NC, USA). The values for maximum tear fluid concentration and its time (C_max_ and t_max_, respectively) as well as the area under the tear fluid concentration-time curve up to 24 h (AUC_0–24_) and mean residence time (MRT) were computed for the tested samples.

#### 2.2.5. Statistical analysis of the obtained results

All results were presented as mean ± SD (standard deviation). The statistical level of significance was set at p-value < 0.05. One-way ANOVA followed by Post Hoc multiple comparisons using LSD test was performed using SPSS^®^ 19.0 software (SPSS Inc., Chicago, IL, USA).

## 3. Results and discussion

### 3.1. Preparation of the nano-spray dried terconazole-loaded Syloid^®^ microparticles

In the preliminary studies, microparticles were prepared using different terconazole: Syloid^®^ weight ratios (2:1, 1:1, 1:2, 1:4 and 1:6 w/w). The prepared microparticles were investigated regarding their Y%, PS, SPAN, DC%, DL%, Q_0.5_ and RE% (Table 1).

All investigated weight ratios produced easily collected microparticles except for the 2:1 weight ratio as the obtained particles were sticky on the nano-spray dryer collector. This might be due to the increased proportion of terconazole relative to Syloid^®^ which masked and diminished the anti-tackling effect of Syloid^®^. Consequently, this ratio was excluded from any further characterization.

### 3.2. Characterization of the nano-spray dried terconazole-loaded Syloid^®^ microparticles

Results presented in Table 1 revealed that the Y%-values for the spray-dried microparticles ranged from 30.69 ± 0.61 to 33.50 ± 0.71% for the microparticles prepared using weight ratio of 1:4 and 1:2 w/w, respectively. Furthermore, the PS-values for the prepared microparticles ranged from 1.82 ± 0.19 to 1.94 ± 0.11 µm for drug: Syloid^®^ weight ratio of 1:1 and 1:6 w/w, respectively. Upon statistically analyzing the obtained results for Y% and PS, all formulations exhibited insignificantly different values from each other (*p* > 0.05) regarding these evaluated parameters.

In addition, SPAN values ranged from 1.29 ± 0.08 to 1.42 ± 0.01 for drug: Syloid^®^ weight ratios of 1:4 and 1:1, respectively. Moreover, the DC% for the formulations ranged from 42.35 ± 0.93% in case of the formulation prepared using drug: Syloid^®^; 1:1 w/w weight ratio to 73.98 ± 1.45% in case of using 1:6 w/w weight ratio. It was observed that the formulation with weight ratio of 1:1 exhibited a significantly lower DC% value as well as significantly higher SPAN-values (*p < 0.05*) compared to the other weight ratios with higher Syloid^®^ content. This can be explained by the fact that increasing the amount of Syloid^®^ (from 1:1 to 1:6 w/w; drug: Syloid^®^) provided more attachment and binding sites for the drug on its surface, consequently an increase in its deposition on the surface of carrier is expected. Hence, in the formulation prepared using ratio of 1:1 (lower Syloid^®^ content), higher SPAN values were obtained which might be ascribed to the insufficiency of the binding sites on Syloid^®^ surface leading to the presence of a higher percentage of unbound drug. Due to the wider variation of the investigated particles sizes, consequently a higher SPAN value was observed (*p* < 0.05).

On the other hand, opposite findings were detected when analyzing the obtained DL% values. The values ranged from 9.75 ± 1.06% to 22.75 ± 0.64% for formulations with 1:6 and 1:2 weight ratios, respectively. It was observed that upon increasing Syloid^®^ amount in the drug: Syloid^®^ weight ratio from 1:1 and 1:2 to 1:4 and 1:6, there was a significant decrease (*p < 0.05*) in DL%. This is in logical accordance with the increase in the total solids relative to a constant drug amount (decrease mass ratio of drug to microparticles) (Zaghloul et al., 2022).

**Figure 1.**
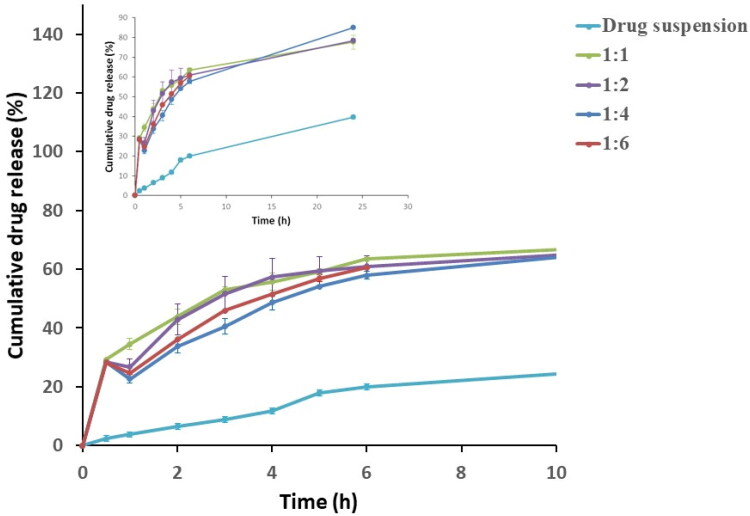
Release profiles of terconazole-loaded Syloid^®^ microparticles during the preliminary studies compared to the drug suspension.

On comparing the release profiles of all the investigated formulations to the corresponding drug suspension, a significant enhancement in the whole release pattern was observed as shown in Figure 1. This proves that incorporation of the mesoporous carrier Syloid^®^ had a tremendous impact on improving the solubility of the poorly-water soluble drug, terconazole (Patel & Dave, 2013).

In addition, Q_0.5_% for drug suspension (2.35 ± 0.13%) was compared to that for evaluated formulations where their Q_0.5_% values ranged from 28.28 ± 0.09% for the formulation prepared using drug: Syloid^®^ weight ratio of 1:6 to 30.32 ± 0.15% for weight ratio of 1:1 as presented in Table 1 and Figure 1. Statistical analysis revealed that formulation prepared using drug: Syloid^®^ weight ratio of 1:1 showed a significantly higher (*p < 0.05*) Q_0.5_ value than the other prepared weight ratios (1:2, 1:4 and 1:6). This increase in Q_0.5_% might be attributed to the increase in the amount of the free drug that failed to upload onto Syloid^®^ resulting in a burst effect. In contrary, the increase in Syloid^®^ content in the other weight ratios presented more room for drug to be uploaded into it, consequently decreasing the initial drug release.

Moreover, RE% from the microparticles ranged from 63.51 ± 0.94% for the formulation prepared using drug: Syloid^®^ weight ratio of 1:4 w/w to 64.88 ± 1.08% for the formulation prepared with weight ratio of 1:1 w/w with no significant difference (*p > 0.05*) between their RE% values.

### 3.3. Preparation of modified microparticles

According to the results obtained from the characterization of drug-loaded Syloid^®^ microparticles during the preliminary studies, and upon applying the desirability function targeting the highest Y%, DC%, DL% and RE% values as well as the lowest SPAN and Q_0.5_ values, it was found that the formulation prepared using drug: Syloid^®^ weight ratio of 1:2 w/w showed the highest desirability value and hence was chosen for the preparation of the modified formulations by adding different percentages of PLGA (Table 2). PLGA with different grades was added aiming at modifying the drug release from the drug-loaded Syloid^®^ microparticles. The obtained results from the characterization of the modified formulations are shown in Table 2.

### 3.4. Characterization of the modified formulations

#### 3.4.1. Nano-spray drying process yield percentage determination

Yield%-values of all the modified formulations ranged from 29.28 ± 1.02 to 54.91 ± 0.13% for SPE_50_-2 and SPA_75_-1, respectively as shown in Table 2. It can be clearly demonstrated that, generally the modified formulations prepared using PLGA with monomers ratio of 75:25 (SPE_75_-1, SPA_75_-1 and SPE_75_-2) had significantly higher yield%-values (*p < 0.05*) than their counterpart formulations prepared using PLGA with monomers ratio of 50:50 (SPE_50_-1, SPA_50_-1 and SPE_50_-2). This might be attributed to the lower melting point of the PLGA with monomers ratio of 50:50 which is around 81 °C *versus* that of PLGA with monomers’ ratio of 75:25 which is in the range of 150-160 °C (Dumitriu & Popa, 2013). Furthermore, it has been reported that the Tg (glass transition temperature) of PLGA decreases with decreasing lactide content (Passerini & Craig, 2001) in the copolymer composition which is found to be 37 °C in case of PLGA with monomers ratio of 50:50 and 42 °C in case of PLGA with monomers ratio of 75:25. This decrease in Tg transforms the polymer to its rubbery state in the collector (outlet temperatures of 40 °C). As a result of using PLGA with lower melting point and Tg-values, melting and adhesion for the microparticles occurs during the spray drying process, resulting in decreased yield %.

On the other hand, formulations prepared using 30% w/w PLGA (SPE_50_-1, SPA_50_-1, SPE_75_-1 and SPA_75_-1) revealed significant higher yield%-values (*p < 0.05*) compared to those prepared using 60% w/w PLGA (SPE_50_-2, SPA_50_-2, SPE_75_-2 and SPA_75_-2). Although this might be surprising but by checking the required time for spraying the same volumes of the investigated samples, it was observed that the spraying process of microparticles containing 30% w/w PLGA exhibited shorter spraying time (around 7 min/10 mL), while the spraying time increased to be between 9 and 11 min upon increasing PLGA concentration to 60% w/w. This longer spraying time required for the higher concentrations of PLGA (regardless its type) was attributed to the associated increase in the viscosity of the feeding solution during the spray-drying process (Huang & Zhang, 2018), which prolonged both the spraying and exposure time of deposited microparticles on the collector. This of course exposed the microparticles to the drying temperature for longer periods of time, resulting in the formation of a sticky yield accounting for losing part of the spray-dried microparticles inside the collector. Furthermore, spraying liquids with high viscosity may cause incomplete solvent evaporation and low Y% (Sansone et al., 2011).

#### 3.4.2. Particle size and SPAN determination

Results of particle size and SPAN measurements of the prepared modified terconazole-loaded Syloid^®^ microparticles are given in Table 2. Results clearly showed that there was no significant impact (*p > 0.05*) of the variation in the PLGA monomers ratio nor the PLGA % on the obtained particle size results as it ranged from 1.711 ± 0.132 to 1.816 ± 0.13 µm for formulation SPE_50_-2 and SPE_75_-1, respectively.

Moreover, the particle size distribution results which was expressed as SPAN showed significant difference (*p < 0.05*) and the obtained values ranged from 1.395 ± 0.014 to 1.569 ± 0.014 for the dried formulation SPA_50_-1 and SPE_75_-2, respectively.

The smaller the SPAN value, the narrower the particle size distribution. To be more precise, a SPAN value of less than 1.5 implies narrow particle size distribution (Merkus, 2009). Generally, most of the prepared formulations had a SPAN value less than 1.5 which is indicative of a narrow distribution profile among all the microparticles population. As shown in Table 2, SPAN results demonstrated that increasing the concentration of PLGA from 30% w/w (SPE_50_-1, SPA_50_-1, SPE_75_-1, SPA_75_-1) to 60% w/w (SPE_50_-2, SPA_50_-2, SPE_75_-2, SPA_75_-2) with both monomers ratios resulted in a significant increase (*p < 0.05*) in SPAN -values. The wider particle size distribution of the microparticles prepared using higher PLGA concentration might be attributed to the increased viscosity of the prepared formulations, which in turn might result in increased adhesion and aggregation during the spray drying process leading to the observed increase in the SPAN values.

#### 3.4.3. Percentage drug content and drug loading determination

DC% was evaluated in all the prepared modified formulations and results were documented in Table 2. In general, DC%-values ranged between 47.23 ± 0.39 and 59.31 ± 0.08% for SPA_75_-2 and SPA_50_-1, respectively. The DC % showed significant reduction (*p < 0.05*) upon increasing the concentration of the used PLGA from 30 to 60% w/w. The rationale behind this reduction might be due to the increase in viscosity of the formulation dispersion associated with the increased PLGA concentration as previously mentioned under Section 3.4.1. This increase in viscosity might form a sticky mass which couldn’t be atomized easily affecting the encapsulation of the drug.

Considering the data presented in Table 2, we can detect that the modified microparticles prepared using PLGA with monomers ratio of 50:50 exhibited higher DC% when compared with their corresponding ones prepared with PLGA with monomers ratio 75:25. This could be attributed to the faster solidification of PLGA with lower Tg (PLGA with 50:50 monomers ratio) which enhance drug content.

Furthermore, it was evaluated that generally the modified formulations made up with the ester capped PLGA showed significant increase (*p < 0.05*) in DC% than their counterpart formulations with the acidic terminal PLGA. This might be attributed to the ability of the COOH group in the acid terminal moiety to form hydrogen bonds with the silanol groups on Syloid^®^ surface which might compete with the drug on the attachment sites on the Syloid^®^ surface leading to a decreased DC%; in other words, decreased host-guest interaction. This is in accordance with the findings of Mellaerts *et al.* (Mellaerts et al., 2011) who reported the hindrance of the co-adsorbed water, due to the formation of hydrogen bonds with silica surface, to the attachment of the drug to the mesoporous silica surface.

Moreover, drug loading results ranged from 11.36 ± 0.08 to 17.14 ± 0.97% for SPA_75_-2 and SPA_50_-1, respectively (Table 2). Results revealed that increasing the concentration of PLGA brought about a significant reduction (*p < 0.05*) in the drug loading percentage. This reduction was expected as the drug concentration was constant in the preparation (5 mg/mL), but the total solid weight increased upon increasing the concentration of PLGA in the formulation.

#### 3.4.4. In-vitro release studies of the modified formulations

**Figure 2.**
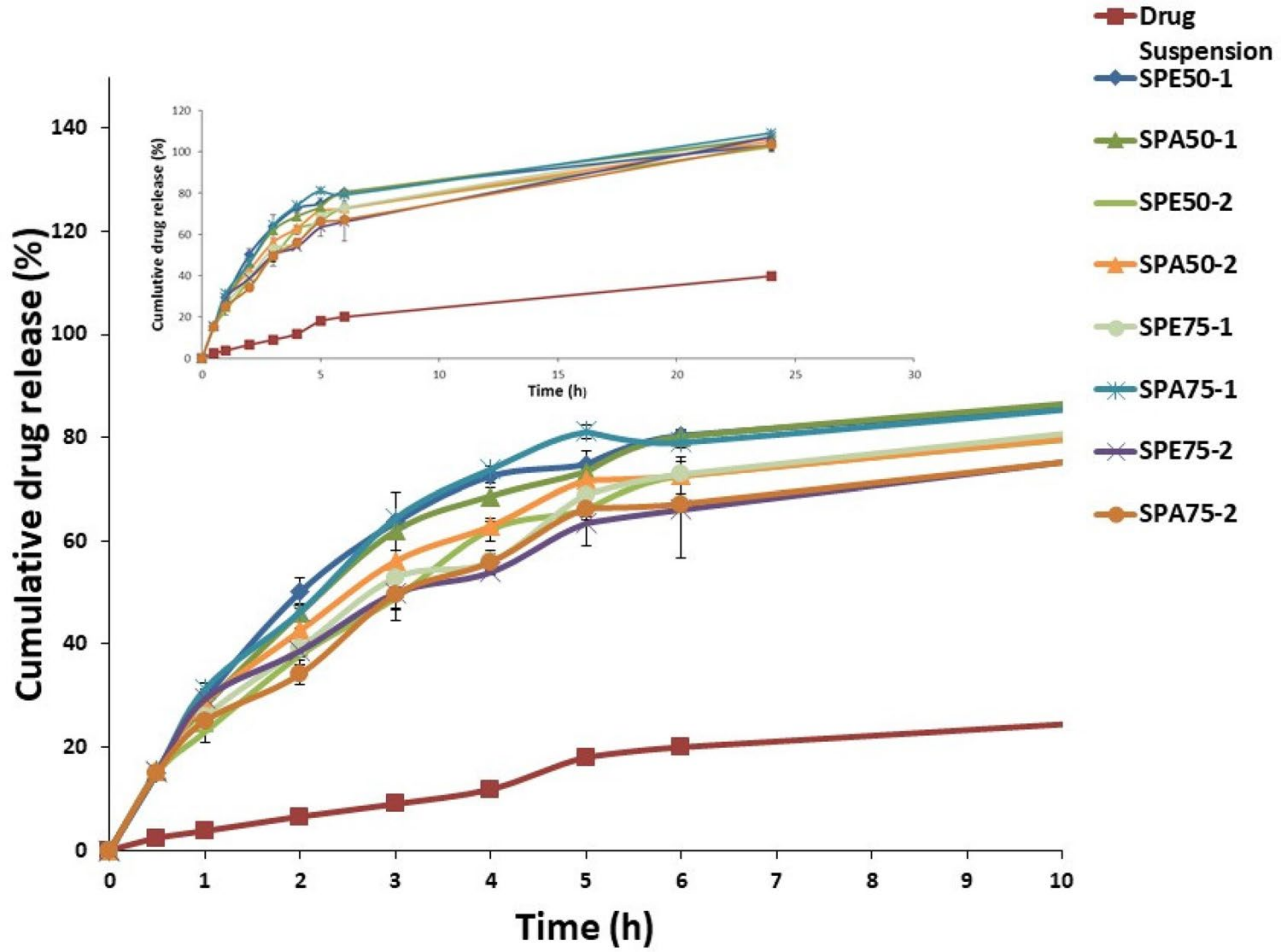
Release profiles of the modified formulations compared to the aqueous drug suspension.

The *in-vitro* release profiles of the modified formulations prepared using different percentages of ester-capped or acid-capped PLGA with different monomers ratios of 50:50 or 75:25 are displayed and compared to terconazole aqueous suspension using the dialysis bag method as presented in Figure 2. All the formulations exhibited faster, and almost complete drug release compared to that for drug suspension.

The modified formulations were assessed by comparing the Q_0.5_ results (Table 2). The obtained results ranged from 15.11 ± 0.11 to 15.85 ± 0.17% for formulation SPE_50_-2 and SPE_75_-2, respectively. After statistical analysis of the obtained results, it was found that the Q_0.5_ were insignificantly different (*p > 0.05*) between all the investigated modified formulations. This proves that neither PLGA monomers ratio and type nor its percentage had a significant effect on the initial release reflected by the results of Q_0.5_. However, upon comparing the obtained Q_0.5_ results of all modified formulations after adding PLGA with that of the optimum drug-loaded Syloid^®^ microparticles prepared during the preliminary studies and lacking the addition of PLGA (drug: Syloid^®^ ratio 1:2; Q_0.5_ = 28.42 ± 0.53%), it was manifested that there was a significant decrease in Q_0.5_ (*p < 0.05*). This highlighted the major effect of PLGA in reducing the burst release of the drug, which might be attributed to the attachment of viscous PLGA locking the attached drug to the surface of Syloid^®^ which have higher tendency to escape causing the burst effect. Additionally, the free drug that failed to attach to Syloid^®^ due to surface saturation might have been entrapped within the matrix of the viscous polymer delaying its release and minimizing the burst release.

Table 2 showed that the increase of concentration of PLGA from 30% w/w (SPE_50_-1, SPA_50_-1, SPE_75_-1 and SPA_75_-1) to 60% w/w (SPE_50_-2, SPA_50_-2, SPE_75_-2 and SPA_75_-2) caused a significant decrease (*p < 0.05*) in % release efficiency. This might be attributed to the concurrent increase of the viscosity of PLGA layer upon increasing PLGA concentration which causes release retardation and subsequently lower release efficiency (Shamma et al., 2017). Furthermore, the rapid solidification of the higher polymer concentration during drying might have better entrapped the free drug that failed to upload on Syloid^®^ surface, thus retarding its release and lowering the overall release efficiency.

Moreover, the modified preparations containing PLGA with ester terminal (SPE_50_-2, SPE_75_-1 and SPE_75_-2) generally showed less release efficiency than their corresponding ones prepared with acidic terminal (SPA_50_-2, SPA_75_-1 and SPA_75_-2). This might be attributed to the hydrophobicity of the ester end group (Konan et al., 2002) in PLGA allowing less water from the release medium to penetrate the polymer matrix and thus slowing down the drug release than PLGA copolymers with terminal carboxylic acid end-groups which possess more hydrophilic character and thus have greater ability for water penetration.

Also, the results revealed that, generally, modified formulations containing PLGA with monomers ratio of 50:50 (SPE_50_-1, SPE_50_-2 and SPA_50_-2) exhibited higher release efficiency than the formulations with monomers ratio of 75:25 (SPE_75_-1, SPE_75_-2 *and* SPA_75_-2). This might be explained by the fact that, lactic acid is more hydrophobic than glycolic acid (Youshia et al., 2017) due to the presence of methyl side groups in it and hence lactide rich PLGA copolymers are less hydrophilic, absorb less water and consequently degrade more slowly, thus presents better retardation and lower RE% (Makadia & Siegel, 2011). Therefore, the modified formulations prepared using PLGA with 50:50 monomers ratio exhibited the fastest degradation and so the highest release efficiency. In addition, the higher molecular weight possessed by PLGA with monomers ratio of 75:25 will give preparations with higher viscosity than the one prepared with PLGA with monomers’ ratio of 50:50. The resulted higher viscosity erupts the release mechanism of the drug from the surrounding polymer reducing the release efficiency from these formulations.

Upon applying the desirability function with the indicated target criteria, it was found that formulation SPA_50_-1 proved superiority in drug content and drug loading and hence was chosen for further physicochemical characterizations, morphological and bioavailability performance investigations.

#### 3.4.5. Differential scanning calorimetry study (DSC)

**Figure 3.**
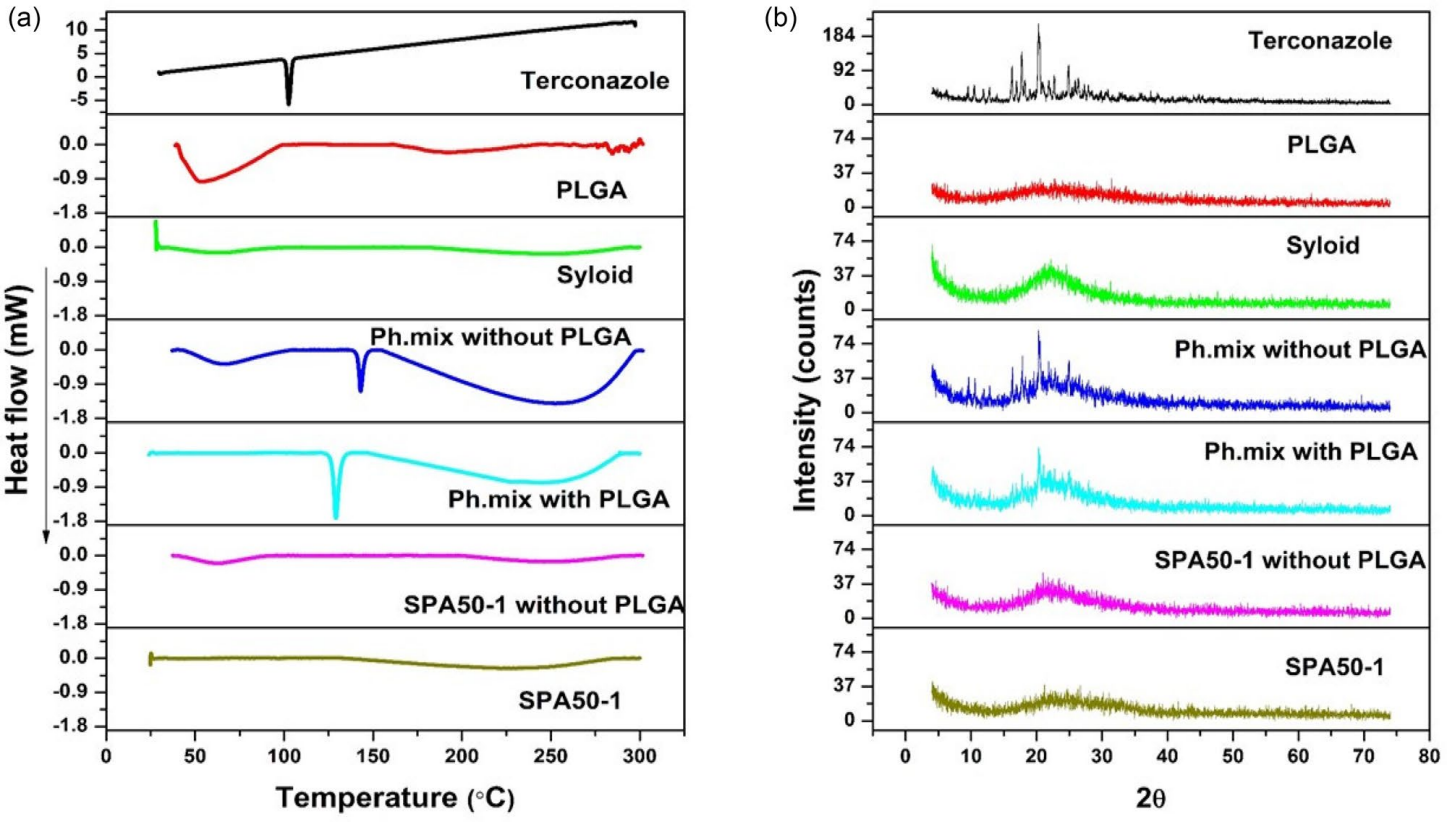
(a) DSC curves and (b) X–ray diffractions for terconazole, PLGA, Syloid^®^, SPA_50_-1 physical mixture without PLGA, SPA_50_-1 physical mixture with PLGA, the selected formulation SPA_50_-1 PLGA-free analogue and the selected formulation SPA_50_-1. Ph.mix: Physical mixture.

Differential scanning calorimetrical analysis was employed to evaluate the thermal behavior of terconazole in the prepared formulation. The DSC thermograms of the chosen modified formulation (SPA_50_-1) and its PLGA-free analogue, as well as its individual components and the corresponding physical mixture are presented in Figure 3a.

The DSC thermogram of pure terconazole showed a characteristic sharp melting endothermic peak at 126.21 °C, corresponding to its melting point, revealing a crystalline anhydrous substance typical behavior. PLGA acid-capped and monomers ratio of 50:50 displayed very broad endothermic peak at 32.65 °C which is around its Tg value.

Both SPA_50_-1 physical mixture and the PLGA-free analogue physical mixture possessed two endothermic peaks at 129.10 versus 129.08 °C, respectively. Those peaks are almost the same peaks appearing in the DSC thermogram of the individual components. The slight shifting in the drug peak might be attributed to the heat used during the thermal analysis. Interestingly, all the peaks of the tested excipients disappeared in the thermogram of the modified formulations; SPA_50_-1 and its PLGA-free analogue, suggesting the complete transformation of the crystalline excipients to the amorphous state including the drug itself due to its potential adsorption on the pores of mesoporous Syloid^®^ particles.

#### 3.4.6. X-ray diffraction study (XRD)

The XRD study was carried out to confirm the results of the DSC studies. Figure 3b illustrates the diffractograms of pure terconazole, PLGA acid-capped and monomers ratio of 50:50, Syloid^®^, SPA_50_-1 and SPA_50_**-**1 PLGA-free analogue as well as their corresponding physical mixtures. It was observed that the pure drug exhibited a diffraction pattern with numerous distinctive peaks indicating its highly crystalline state. The most abundant peak was observed at 2θ values of 20.31°.

The diffraction patterns of PLGA showed no characteristic peaks indicating its amorphous state. However, Syloid^®^ showed one broad peak at 2θ values of 22.21°. The XRD profile of the physical mixtures for SPA_50_-1 and SPA_50_-1 PLGA-free analogue also demonstrated the crystalline peaks for terconazole corresponding to 2θ values of 20.39° and 20.37°, respectively. As well as the broad peak for Syloid^®^. However, the terconazole peak intensity was lessened. This might be due to the relative decrease in the drug concentration in the physical mixture in comparison to the pure ingredients.

The selected formula SPA_50_-1 and its PLGA-free analogue showed one broad typical halo pattern and the peaks attributable to the crystalline terconazole disappeared. This indicates the entirely amorphous nature of terconazole in the system and its homogenous distribution in Syloid^®^ in its amorphous state. This confirms the results obtained with the DSC study.

#### 3.4.7. Scanning electron microscopy (SEM)

**Figure 4.**
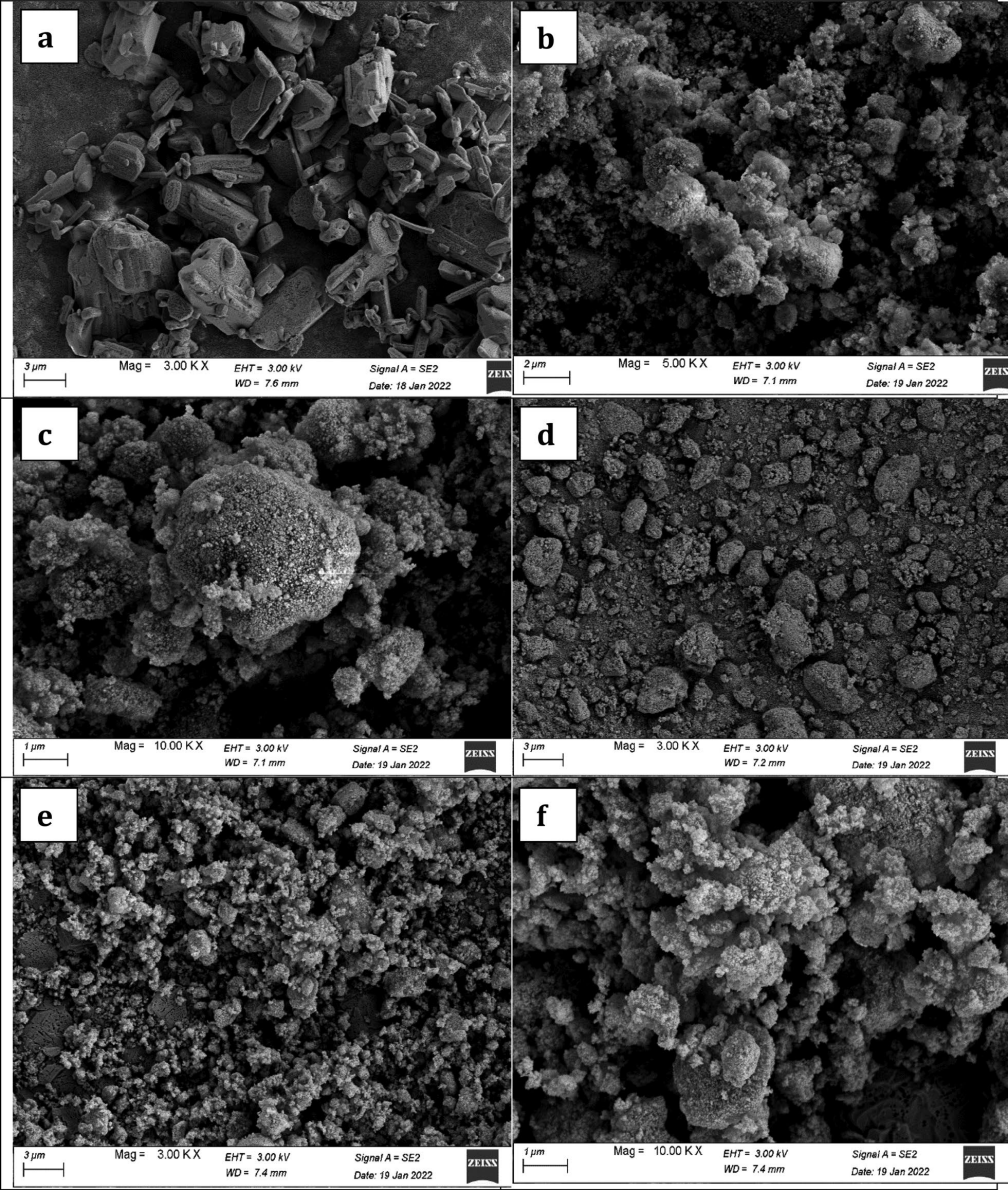
SEM images of (a) Terconazole, (b & c) Syloid^®^, (d) SPA_50_-1 PLGA-free analogue and (e & f) SPA_50_-1.

SEM micrographs of the pure drug (terconazole), the selected modified formulation SPA_50_-1, its PLGA-free analogue and pure Syloid^®^ are shown in Figure 4. The micrographs of the pure drug showed rectangle crystals which disappeared in the micrographs of the prepared formulation in the presence of Syloid^®^ with or without PLGA. This proved the complete loading of drug onto Syloid^®^. Moreover, the micrographs of formulations prepared with or without PLGA retained the rough and porous surface for Syloid^®^ which proves that the PLGA was embedded and attached on Syloid^®^.

#### 3.4.8. Biological evaluation of the optimized formulation

##### 3.4.8.1. Histopathological study

Ocular products, must be well tolerated by the eyes and should not cause any discomfort to the patients. Inadequate patient use of an ocular medication is a major issue, owing to low clinical acceptance caused by unpleasant side effects like irritation, burning, stinging, and ripping (Maulvi et al., 2021).

**Figure 5.**
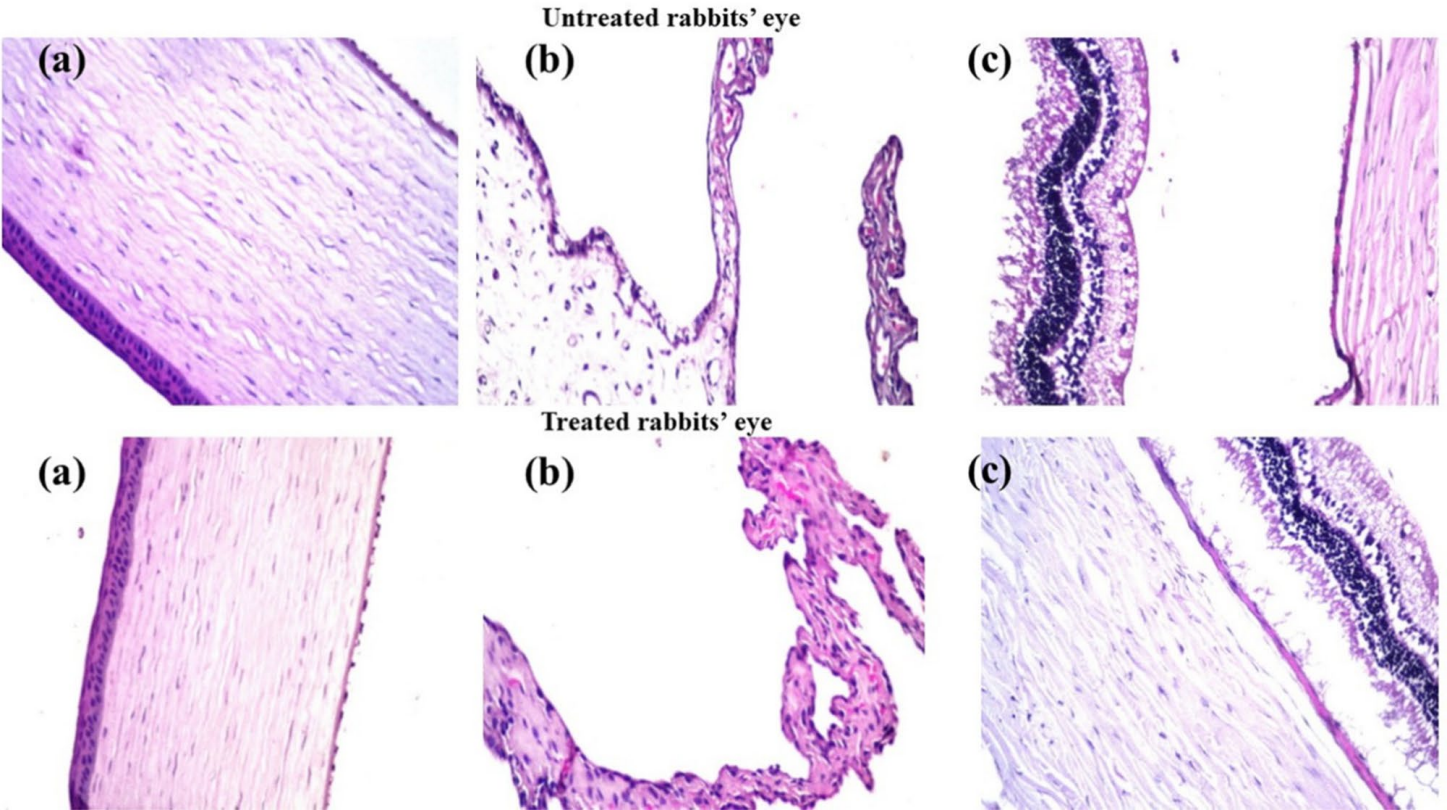
Photomicrographs introducing histopathological sections of normal untreated rabbit eye and rabbit eye treated with optimum SPA_50_-1, showing histological structure of the (a) cornea, (b) iris, (c) retina, choroid and sclera. All with magnification power of 40x.

Therefore, histopathological studies were carried out on ocular tissues of male Albino rabbits. The samples were stained by hematoxylin and eosin stain for light electric microscope examination of both groups (group I: untreated rabbit eye and group II: SPA_50_-1 treated rabbit eye). Group II showed normal histological structure with no histopathological retardation in the cornea, iris, retina, choroid and sclera as shown in Figure 5.

This evidence proved the safety profile of the used components in the preparation of the selected modified formulation for the ocular use.

##### 3.4.8.2. Pharmacokinetic study

**Figure 6.**
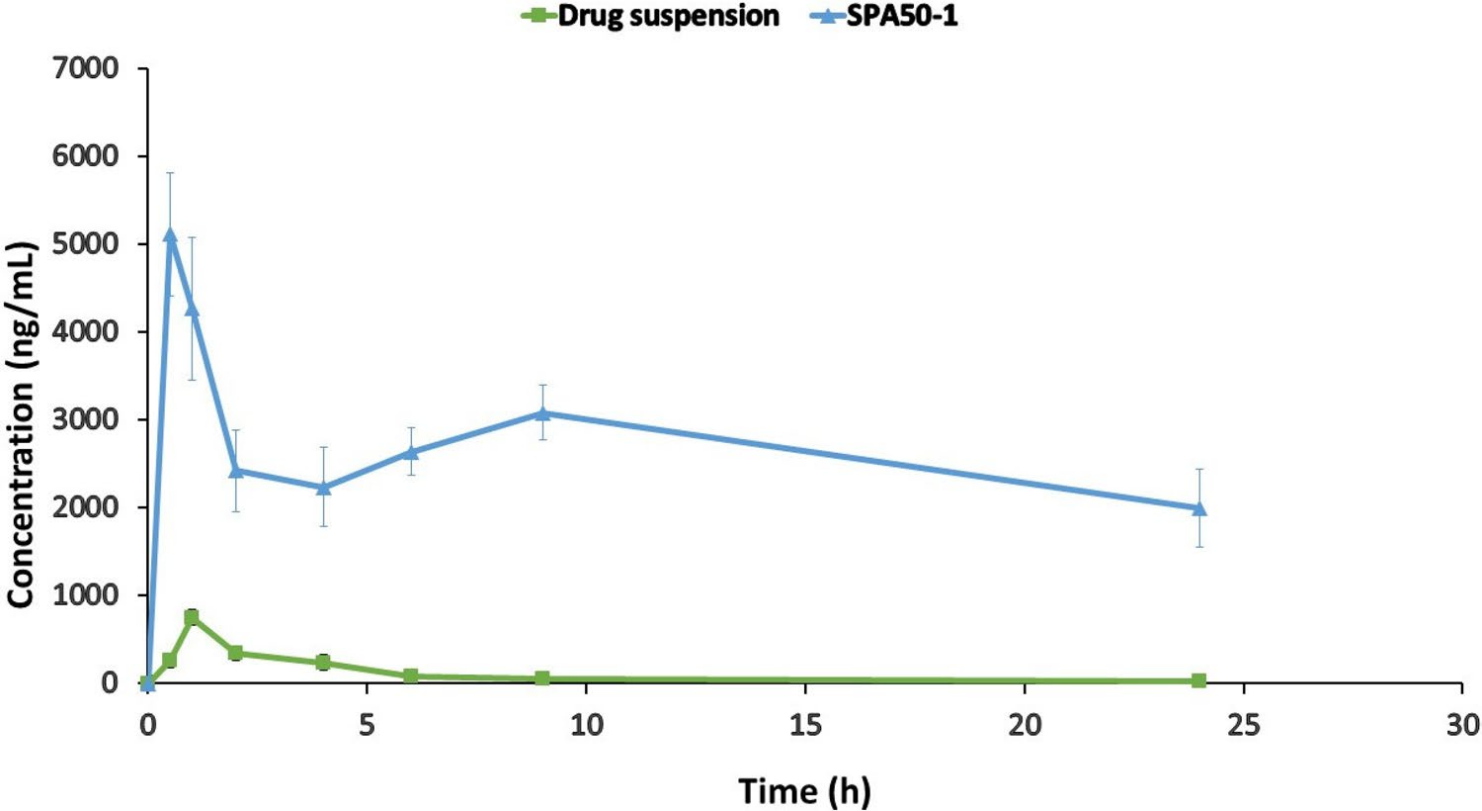
Teraconazole concentrations in rabbits’ tears-time curve for the investigated formulation compared to the drug suspension.

The pharmacokinetic parameters of terconazole were evaluated for the selected formulation SPA_50_-1 and compared to the drug suspension after dosing in rabbits’ eyes at a concentration of 1 mg/mL. Table 3 represents the pharmacokinetic parameters of the administered samples. Additionally, Figure 6 shows completely different tear concentration/time curve profiles with the superiority of the examined formulation (*p < 0.05*) over the drug suspension regarding the C_max_, t_max_, AUC_0–24_ and MRT. The C_max_ values for formulation SPA_50_-1 and drug suspension were 5112.5 ± 705.35 and 752.19 ± 81.17 ng/mL achieved after 0.5 and 1 h (t_max_), respectively. Moreover, AUC_0–24_ values were found to be 63141.75 ± 5698.24 and 2494.07 ± 274.02 ng.h/mL for SPA_50_-1 and drug suspension, respectively. Regarding the C_max_ and AUC_0–24_ values, it was found that 6.7 and 25.3-fold increase, respectively were obtained with formulation SPA_50_-1 compared to the drug suspension. Also, MRT values were found to be 4.90 ± 0.30 and 10.69 ± 0.63 h for the drug suspension and SPA_50_-1, respectively. Higher value of MRT ensured the ability of the modified formulation SPA_50_-1 to extend the residence of the drug on the ocular tissues compared to the drug suspension. This extended MRT results might be related to the sustained *in-vitro* release profile of the modified formulation. In conclusion, the incorporation of terconazole into the selected formulation SPA_50_-1 succeeded to enhance the drug ocular bioavailability.

**Table 3.** Pharmacokinetic parameters of the selected PLGA-modified Syloid^®^ microparticles compared to the drug suspension.

Pharmacokinetic parameter	Drug suspension	SPA_50_-1
**C_max_ (ng/mL)**	752.2 ± 81.2	5112.5 ± 705.3
**t_max_ (h)** *	1.0	0.5
**AUC (ng h/mL)**	2494.1 ± 274.0	63141.7 ± 5698.2
**MRT (h)**	4.9 ± 0.3	10.6 ± 0.6

* Values represent median.

## 4. Conclusion

Spray-dried terconazole-loaded Syloid^®^ microparticles were successfully prepared for the ocular use. Moreover, sustained release performance was achieved *via* attaching the selected formulation with PLGA polymer in different concentrations. The modified microparticles prepared using 30%w/w acid-capped PLGA with monomers ratio 50:50 was proved to have the highest drug content %, drug loading % and release efficiency % as well as the lowest SPAN and % drug release after 0.5 h. Upon comparing the release profile as well as the pharmacokinetic parameters of the selected formulation to the drug suspension, the formulation proved to be superior. The C_max_ and AUC_0–24_ -values for the selected formulation were 6.7 and 25.3-fold higher than those obtained for the drug suspension. Also, higher MRT values were observed which ensured the ability of the selected formulation to extend the residence of the drug on the ocular tissues compared to the drug suspension. Moreover, the ocular safety profile of the investigated formulation was proved. These results evidenced the potentiality of the prepared formulation to augment both the release profile and the ocular bioavailability of the drug.

## Data Availability

Data available within the article.
